# Type IV Pili-Independent Photocurrent Production by the Cyanobacterium *Synechocystis* sp. PCC 6803

**DOI:** 10.3389/fmicb.2020.01344

**Published:** 2020-06-25

**Authors:** Miyuki A. Thirumurthy, Andrew Hitchcock, Angelo Cereda, Jiawei Liu, Marko S. Chavez, Bryant L. Doss, Robert Ros, Mohamed Y. El-Naggar, John T. Heap, Thomas S. Bibby, Anne K. Jones

**Affiliations:** ^1^School of Molecular Sciences, Arizona State University, Tempe, AZ, United States; ^2^Department of Molecular Biology and Biotechnology, The University of Sheffield, Sheffield, United Kingdom; ^3^Department of Physics, Arizona State University, Tempe, AZ, United States; ^4^Department of Physics and Astronomy, University of Southern California, Los Angeles, CA, United States; ^5^Department of Biological Sciences, University of Southern California, Los Angeles, CA, United States; ^6^Department of Chemistry, University of Southern California, Los Angeles, CA, United States; ^7^Imperial College Centre for Synthetic Biology, Department of Life Sciences, Imperial College London, London, United Kingdom; ^8^School of Life Sciences, University of Nottingham, Nottingham, United Kingdom; ^9^Ocean and Earth Science, University of Southampton, Southampton, United Kingdom

**Keywords:** cyanobacteria, type IV pili, nanowire, photocurrent, biophotovoltaics, extracellular electron transfer

## Abstract

Biophotovoltaic devices utilize photosynthetic organisms such as the model cyanobacterium *Synechocystis* sp. PCC 6803 (*Synechocystis*) to generate current for power or hydrogen production from light. These devices have been improved by both architecture engineering and genetic engineering of the phototrophic organism. However, genetic approaches are limited by lack of understanding of cellular mechanisms of electron transfer from internal metabolism to the cell exterior. Type IV pili have been implicated in extracellular electron transfer (EET) in some species of heterotrophic bacteria. Furthermore, conductive cell surface filaments have been reported for cyanobacteria, including *Synechocystis*. However, it remains unclear whether these filaments are type IV pili and whether they are involved in EET. Herein, a mediatorless electrochemical setup is used to compare the electrogenic output of wild-type *Synechocystis* to that of a Δ*pilD* mutant that cannot produce type IV pili. No differences in photocurrent, i.e., current in response to illumination, are detectable. Furthermore, measurements of individual pili using conductive atomic force microscopy indicate these structures are not conductive. These results suggest that pili are not required for EET by *Synechocystis*, supporting a role for shuttling of electrons via soluble redox mediators or direct interactions between the cell surface and extracellular substrates.

## Introduction

Electron transfer and redox reactions form the foundation for energy transduction in biological systems (Marcus and Sutin, 1985). Some microbes have the capacity to transfer electrons beyond their cell wall to extracellular acceptors (Hernandez and Newman, 2001), a function that may be important in microbial ecology (Lis et al., 2015; Polyviou et al., 2018) and has been exploited in bioelectronic applications. Although electron transfer between redox-active sites separated by less than 1.6 nm is well understood to occur via electron tunneling described by Marcus theory, little is known about the mechanisms of electron transfer over larger distances, i.e., nanometers to micrometers, observed in biological ecosystems (Gray and Winkler, 2005). Long-range electron transfer in various microbes may employ soluble redox mediators, conductive bacterial nanowires or pili (Reguera et al., 2005; Marsili et al., 2008; Brutinel and Gralnick, 2012; Kotloski and Gralnick, 2013; Yang et al., 2015; Ing et al., 2018; Heidary et al., 2020). Furthermore, an understanding of this activity forms the foundation for the development of microbial fuel cells and photobiological electrochemical systems, devices that employ microbes to generate electricity (Rabaey and Verstraete, 2005; Kracke et al., 2015).

Two distinct mechanisms have been hypothesized to account for extracellular electron transfer (EET) in anaerobic, heterotrophic bacteria: utilization of soluble, diffusing redox shuttles like flavins to transfer electrons from the cellular interior to the extracellular surface (Watanabe et al., 2009; Glasser et al., 2017) and direct interaction between a redox-active component on the cell surface and the extracellular target (Shi et al., 2009). The latter has been proposed to proceed via redox proteins on the cell surface (e.g., multiheme cytochromes) or via extracellular appendages that have come to be known as bacterial nanowires (Gorby et al., 2006; El-Naggar et al., 2010). The composition of these nanowires is hypothesized to vary between different organisms; recent work by El-Naggar and coworkers has shown that the nanowires of *Shewanella oneidensis* MR-1 are extensions of EET-protein-containing outer membrane that appear to form from chains of vesicles (Pirbadian et al., 2014). On the other hand, Lovley and coworkers reported that the nanowires of electrogenic *Geobacter* sp. are conductive pili (Reguera et al., 2005; Holmes et al., 2016), whereas recent studies have shown that *Geobacter sulfurreducens* produces OmcS cytochrome filaments that are distinct from type IV pili (Tfp) (Filman et al., 2019; Wang et al., 2019). For a recent review of *Geobacter* protein nanowires see Lovley and Walker (2019). However, details about the types of charge carriers and the exact mechanisms of interfacial electron transport within conductive appendages remain unclear.

Biophotovoltaic devices (BPVs) interconvert light and electrical energy using a photosynthetic organism. The most common devices employ oxygenic phototrophs to harvest light energy and transfer electrons produced by water oxidation to extracellular acceptors, generating power or hydrogen (Zou et al., 2009; Pisciotta et al., 2010; McCormick et al., 2011, 2015; Bradley et al., 2012; Lea-Smith et al., 2015; Saper et al., 2018; Tschörtner et al., 2019). Cyanobacteria, green algae, and plants have been used to generate power in BPVs, with much work performed using the model freshwater cyanobacterial species *Synechocystis* sp. PCC 6803 (hereafter *Synechocystis*). Current production in BPVs containing *Synechocystis* is largely dependent on illumination, and previous studies employing chemical and genetic inhibition indicate that water splitting by Photosystem II (PSII) provides the majority of electrons (Bombelli et al., 2011; Pisciotta et al., 2011; Cereda et al., 2014). Improvements of BPVs based on advances in device architecture, electrode material, proton exchange membrane and use of mediators and biofilms have been reported (Thorne et al., 2011; Bombelli et al., 2012, 2015; Call et al., 2017; Rowden et al., 2018; Wenzel et al., 2018; Wey et al., 2019), but improvements arising from engineering of phototrophs have been limited to genetic removal of competing electron sinks (Bradley et al., 2013; McCormick et al., 2013; Saar et al., 2018) by lack of understanding of how photosynthetic electrons are transferred from the photosynthetic apparatus to extracellular acceptors.

Tfp are required for gliding motility, phototaxis, cell adhesion, flocculation, and natural transformation competency in *Synechocystis*, which produces morphologically distinct thick (∼5–8 nm, >2 μm in length, form tufts) and thin (∼3–4 nm, ∼1 μm, cover whole surface of cell) pili (Bhaya et al., 2000; Yoshihara et al., 2001; Schuergers and Wilde, 2015; Chen et al., 2020). Tfp have also been implicated as having a role in reductive iron (Kranzler et al., 2011; Lamb et al., 2014) and manganese uptake (Lamb and Hohmann-Marriott, 2017). *Synechocystis* has also been reported to produce conductive filaments under conditions of CO_2_ limitation (Gorby et al., 2006), although whether these are Tfp is unclear. For detailed reviews of Tfp structure, biogenesis, and function in *Synechocystis*, see Schuergers and Wilde (2015) and Chen et al. (2020).

*Synechocystis* cannot produce pili in the absence of the leader peptidase/methylase, encoded by the *pilD* gene (Bhaya et al., 2000). Herein, the rates of EET by a Δ*pilD* mutant are compared to those of wild-type organisms by measuring photocurrent production in our previously described mediatorless bioelectrochemical cell (Cereda et al., 2014). Photocurrent production by the wild-type and Δ*pilD* cells is not significantly different, suggesting pili do not play a role in photocurrent generation or EET by *Synechocystis*, at least under the conditions investigated here. Additionally, conductivity measurements using atomic force microscopy (AFM) of wild-type *Synechocystis* pili found no evidence for conductivity in these structures. Our results support the hypothesis that redox mediator shuttling may be the major mechanism of photocurrent production by cyanobacteria (Saper et al., 2018; Wenzel et al., 2018).

## Materials and Methods

### Growth of *Synechocystis* sp. PCC 6803

A glucose-tolerant (GT) strain of *Synechocystis* was used as the wild type in this study (see Supplementary Table S1 for details). *Synechocystis* was cultured in BG11 media (Rippka et al., 1979) buffered with 10 mM N-[tris(hydroxymethyl) methyl]-2-aminoethanesulfonic acid (TES)-KOH pH 8.2 (BG11-TES). For photoautotrophic growth, 200 ml cultures contained within 250 ml flasks were bubbled with sterile air at 30°C under a constant illumination of approximately 50 μmol photons m^–2^ s^–1^. For photomixotrophic growth, 5 mM glucose was added to the medium. For growth on plates, media was supplemented with 1.5% (w/v) agar and 0.3% (w/v) sodium thiosulphate; 34 μg/ml chloramphenicol (for Δ*pilD*) or 20 μg/ml zeocin (Δ*psbB*) were added where required. Growth was monitored by measurement of the optical density at 750 nm (OD_750_).

### Deletion of *pilD* (slr1120)

For deletion of *pilD*, the central portion of the slr1120 open reading frame was replaced with a chloramphenicol acetyl transferase (*cat*) gene by allele exchange using a plasmid (pICJH4) constructed by Gibson assembly (Gibson et al., 2009) of three PCR products (two amplified from *Synechocystis* genomic DNA and the third from pACYC184) together with the 2.6 kb *Eco*RI*–Hin*dIII restriction fragment of pUC19. The allele exchange cassette comprised a first region of 685 bp of homology with the *Synechocystis* chromosome including upstream flanking sequence and the first 28 codons of *pilD* followed by two stop codons (amplified with primers *pilD*-us-F and *pilD*-us-R), the *cat* cassette (amplified with primers *cat*-F and *cat*-R), and a second region of 500 bp of homology with the *Synechocystis* chromosome beginning with the 12th-from-last codon of *pilD* followed by flanking downstream DNA (amplified with primers *pilD*-ds-F and *pilD*-ds-R) (see Supplementary Table S2 for primer sequences). The pICJH4 plasmid was confirmed to be correctly assembled by automated DNA sequencing and introduced into wild-type *Synechocystis* by natural transformation. Recombinants were selected on plates containing 5 μg ml^–1^ chloramphenicol, and segregation of genome copies was achieved by sequentially increasing the chloramphenicol concentration (up to 40 μg ml**^–1^**). Segregation at the *pilD* locus was confirmed by PCR with primer pair *pilD*-screen-F and *pilD*-screen-R.

### RNA Isolation and RT-PCR

End-point reverse transcriptase PCR analysis of *Synechocystis* strains was performed as described previously for *Acaryochloris marina* (Chen et al., 2016). Briefly, *Synechocystis* cells were harvested at mid-log phase (OD_750_ = ∼0.6), and total RNA was isolated by the hot TRIzol method (Pinto et al., 2009). RNA was treated with the Ambion Turbo DNA-*free*^TM^ Kit to remove contaminating genomic DNA, and 100 ng was used for cDNA synthesis and PCR, which were performed in a single reaction using the MyTaq one-step reverse transcription-PCR (RT-PCR) kit (Bioline). Gene-specific primer pairs *pilA1*-RT-F/R, *pilD*-RT-F/R or *rnpB*-RT-F/R were used to detect transcript of *pilA1* (124 bp), *pilD* (180 bp), and the reference gene *rnpB* (180 bp) (Polyviou et al., 2015), respectively. The reaction setup and thermocycling conditions were performed according to the manufacturer’s instructions, and 10 μl of PCR product was analyzed on a 2% (w/v) agarose gel.

### Immunodetection of PilA1

Denatured whole-cell extracts were separated by SDS-PAGE on 12% Bis-Tris gels (Invitrogen) and transferred to polyvinylidene difluoride membranes (Invitrogen). Membranes were incubated with an anti-PilA1 primary antibody raised against a synthetic peptide corresponding to PilA1 residues 147–160 as described previously (Linhartová et al., 2014) and then a secondary antibody conjugated with horseradish peroxidase (Sigma Aldrich). Chemiluminescence was detected using the WESTAR^®^ EtaC kit (Geneflow Ltd.) and an Amersham^TM^ Imager 600 (GE Healthcare).

### Oxygen Evolution and Determination of Chlorophyll Content

Oxygen evolution was measured as described in our previous work (Cereda et al., 2014). Chlorophyll was extracted from cell pellets from 1 ml of culture at OD_750_ ≈ 0.4 with 100% methanol and quantified spectrophotometrically according to Porra et al. (1989).

### Electrochemical Measurements

Electrochemical measurements were made in a three-electrode cell with carbon cloth as working electrode as described previously (Cereda et al., 2014).

### Atomic Force Microscopy Imaging of Wild-Type and Mutant Cells (Δ*pilD*^∗^)

*Synechocystis* wild-type and Δ*pilD*^∗^ cells grown photoautotrophically in liquid BG11 or on BG11 agar plates were collected, washed three times, and resuspended in 1 ml deionized water (centrifugation speed 3,500 × *g*). Aliquots of 5 μl were spotted onto a mica support and air dried. After drying, samples were imaged using an Asylum Research MFP 3D (Santa Barbara, CA, United States) Atomic Force Microscope (AFM) in tapping mode using Tap300Al-G probes (with 40 N/m force constant, 300 kHz resonant frequency). The images were processed using Gwyddion software.

### Scanning Electron Microscopy (SEM) Imaging

Wild-type *Synechocystis* and the Δ*pilD*^∗^ strain were grown photoautotrophically and harvested via centrifugation (3,500 × *g*). Cells were transferred to the carbon cloth used for electrochemical measurements, fixed onto the cloth in 50 mM sodium phosphate buffer (pH 7.2) with 2% glutaraldehyde for 30 min at room temperature, and washed three times in the same buffer for a total of 30 min. After a second fixation step for 30 min at room temperature in the same buffer plus 0.5% (v/v) osmium tetroxide, samples were washed three times with deionized water. Samples were critical point dried with carbon dioxide (Balzers CPD020 unit), mounted on aluminum specimen stubs, and coated with approximately 15 nm of gold-palladium (Technics Hummer-II sputter-coater). Sample analysis was performed with a JEOL JSM-6300 SEM operated at 15 kV, and images were acquired with an IXRF Systems digital scanning unit.

### AFM-Based Electrical Characterization of Pili

Glass coverslips (43 × 50 NO. 1 Thermo Scientific Gold Seal Cover Glass) coated with 5 nm titanium and then 100 nm gold via electron beam evaporation were used as conductive substrates. The Au-coated coverslips were rinsed with acetone, isopropanol, ethanol, and deionized water and then dried with nitrogen prior to use. *Synechocystis* cells were drop cast onto the clean conductive substrates, rinsed with sterile water, and left to dry overnight. An Oxford Instruments Asylum Research Cypher ES AFM was used to make all electrical measurements. Dried samples were affixed and electrically connected to AFM disks with silver paint (TED PELLA, Inc). The sample disks were wired to the AFM upon loading. Si probes, with a Ti/Ir (5/20) coating, a resonant frequency of 75 kHz (58-97), a spring constant of 2.8 N/m (1.4-5.8), and a tip radius of 28 ± 10 nm, were used (Oxford Instruments AFM probe Model: ASYELEC.01-R2). Pili electrical characterization was performed using Oxford Instruments Asylum Research Fast Current Mapping (FCM). To generate FCM images, a bias is held between the probe and substrate while, for each pixel, current and force are measured with respect to the vertical distance of consecutive probe approaches and retractions over the sample. Each approach is terminated when a user-defined force is met (a force setpoint), and each retraction is terminated when a user-defined distance is met (a force distance). A bias of 5.00 V was used. A force setpoint of 49.34 nN and a force distance of 1000 nm were used for thick pili measurements. A force set point of 27.86 nN and a force distance of 750 nm were used for thin pili measurements.

## Results

### Generation and Phenotypic Analysis of a Δ*pilD* Strain

**FIGURE 1:**
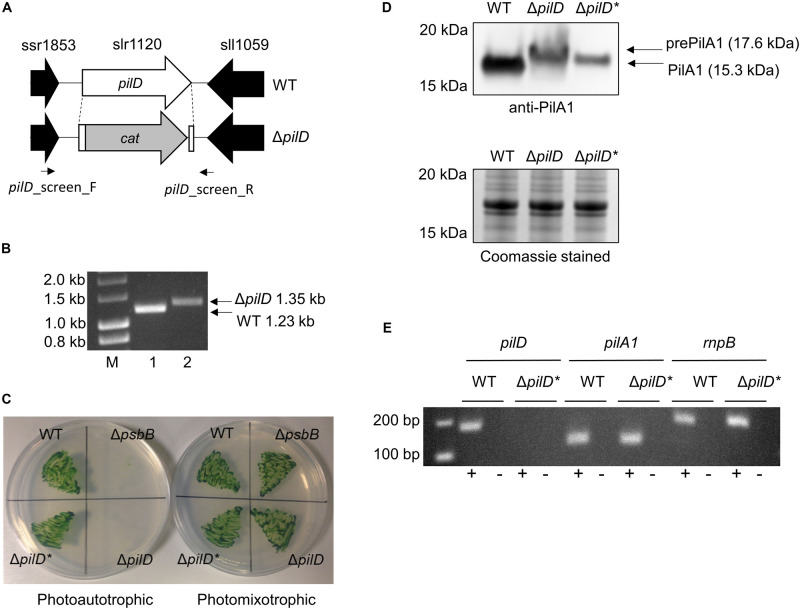
Generation and phenotypic analysis of a Δ*pilD* mutant strain of *Synechocystis*. **(A)** Strategy for deletion of *pilD* (slr1120) by replacement with the chloramphenicol acetyl transferase (*cat*) cassette. The position of screening primers used in panel **(B)** is shown. **(B)** Agarose gel showing PCR products amplified with the *pilD*_screen_F/*pilD*_screen_R primer pair with wild type (WT, lane 1) or Δ*pilD* (lane 2) genomic DNA as template. A larger 1.35 kb PCR product is observed for the Δ*pilD* mutant compared to the 1.23 kb WT band. Lane M = HyperLadder^TM^ 1 kb molecular weight marker (Bioline). **(C)** Growth of the WT, Δ*pilD* and ΔpilD* (suppressor mutant capable of photoautotrophic growth) in the absence or presence of 5 mM glucose. The originally isolated Δ*pilD* mutant cannot grow under photoautotrophic conditions; a Δ*psbB* mutant that is also unable to grow photoautotrophically is included as a control. **(D)** Level of (pre)PilA1 in WT, Δ*pilD* and ΔpilD* in photomixotrophically grown whole-cell extracts determined by immunodetection with anti-PilA1 antibodies (upper panel). The accumulation of prePilA1 in the original mutant is decreased in the suppressor strain. The predicted molecular weights of pre- and processed PilA1 are indicated. The lower panel shows a duplicate Coomassie-stained SDS-PAGE gel to demonstrate approximately equal protein loading of each sample. **(E)** End-point RT-PCR analysis of *pilA1* expression in WT and ΔpilD* showing the transcript is present in both strains. As expected, *pilD* transcripts were absent from the mutant; the *rnpB* housekeeping gene is included as a control. Reactions were performed in the presence (+) or absence (–) of reverse transcriptase.

The PilD protein is a bifunctional, membrane-bound leader peptidase/methylase that processes PilA precursors and N-methylates the amino acid at position 1 in the mature protein (Strom et al., 1993). PilD is absolutely required for pilus assembly, and a Δ*pilD* mutant in a motile strain of *Synechocystis* has been reported to be non-piliated, non-motile, and recalcitrant to transformation (Bhaya et al., 2000). Since *Synechocystis* contains multiple *pilA* genes (Yoshihara et al., 2001) but only a single copy of *pilD* (slr1120), we used a Δ*pilD* knockout mutant to investigate whether pili are required for EET in *Synechocystis*. The Δ*pilD* mutant generated herein has most of the open reading frame replaced with a chloramphenicol resistant cassette (Figure 1A) and was confirmed to be fully segregated by PCR (Figure 1B).

It should be noted that GT strains of *Synechocystis* are typically non-motile because of a frameshift mutation in the *spkA* (sll1574) gene, which in motile strains encodes a functional Ser/Thr protein kinase (Kamei et al., 2001). In the originally genome-sequenced Kazusa strain (Kaneko et al., 1996), a 1 bp insertion also results in a frameshift mutation in *pilC* (slr0162/3), preventing pilus assembly (Bhaya et al., 2000), which means this strain is non-competent for transformation with exogenous DNA (Ikeuchi and Tabata, 2001). The *pilC* mutation seems to be specific to the Kazusa strain as other GT strains contain an intact *pilC* gene (Tajima et al., 2011; Kanesaki et al., 2012; Trautmann et al., 2012; Morris et al., 2014; Ding et al., 2015), and the GT wild-type strain used in this study (Supplementary Table S1) is naturally transformable and thus must produce Tfp.

When first generated, the Δ*pilD* mutant displayed an obvious aggregation phenotype, with cells forming small clumps when grown photoheterotrophically in liquid medium. The cells were very difficult to collect with a loop from an agar plate, and the strain grew very poorly, if at all, under photoautotrophic conditions (Table 1 and Figure 1C). Similar phenotypes were described for a Δ*pilD* mutant generated by Linhartová et al. (2014), who showed that the build-up of unprocessed PilA-prepilins triggered degradation of the essential membrane proteins SecY and YidC. Linhartová et al. (2014) isolated suppressor mutants that were able to grow photoautotrophically by prolonged growth in the absence of glucose or targeted deletion of the *pilA1* gene. Similarly, after continued sub-culturing on agar plates we also isolated suppressor mutants that were capable of photoautotrophic growth, and when cultures were well mixed by air bubbling or orbital shaking, these suppressor strains grew at rates comparable to the wild type without significant clumping (Table 1 and Figure 1C). We will henceforth refer to the strain which can grow photoautrophically as Δ*pilD*^∗^. Linhartová et al. (2014) showed that the loss of PilA1 pre-pilins in their Δ*pilD*^∗^ strain was at least partially responsible for the improvement in growth; conversely, we found that Pre-PilA1 is still present in our Δ*pilD*^∗^strain, albeit to a lesser extent than in the originally isolated Δ*pilD* strain (Figure 1D). Another study found that the level of *pilA1* mRNA in a Δ*pilD* strain capable of phototrophic growth is similar to that of the wild-type organism (Bhaya et al., 2000); sequencing confirmed *pilA1* and its promoter are not mutated in our Δ*pilD*^∗^ strain, and we confirmed *pilA1* is expressed using end-point RT-PCR (Figure 1E), indicating that reduced transcription of the *pilA1* gene is unlikely to be the reason for the decrease in PilA production. Further investigation of the nature of the suppressor mutation(s) in Δ*pilD*^∗^ strains is beyond the scope of the present work and will be reported elsewhere (Linhartova, Sobtoka, et al. Unpublished).

**TABLE 1:** Growth rate, chlorophyll content, and oxygen evolution of WT, Δ*pilD* and ΔpilD* Synechocystis cells.

**Strain**	**Growth condition^a^**	**Doubling time (h)**	**Chl content (μg OD_750_ unit**^–1^)	**O_2_ evolution (nmol O_2_ OD_750_ unit**^–1^** min**^–1^)	**O_2_ evolution (μmol O_2_ mg chl**^–1^** h**^–1^**)^b^**
WT	PM	12 ± 0.5	3.9 ± 0.3	41 ± 1	631
WT	PA	16 ± 0.5	4.2 ± 0.2	46 ± 5	657
Δ*pilD*	PM	20 ± 1.0^c^	3.5 ± 0.7^c^	32 ± 8^c^	549^c^
ΔpilD*	PM	12 ± 0.5	3.8 ± 0.4	40 ± 4	632
ΔpilD*	PA	16 ± 0.5	4.1 ± 0.1	46 ± 4	673

*^*a*^Growth under PM, photomixotrophic (plus 5 mM glucose) or PA, photoautotrophic conditions, as described in the section “Materials and Methods.” ^*b*^Calculated from Chl content of 1 OD_750_ unit of cells and the oxygen evolution (nmol O_2_ OD_750_ unit^–1^ min^–1^). ^*c*^Accuracy of the growth rate, Chl content, and oxygen evolution is limited for this strain as a result of the clumping phenotype.*

The initially isolated Δ*pilD* mutant described by Linhartová et al. (2014) had impaired PSII activity. Because it has previously been shown that photocurrent from *Synechocystis* is largely dependent on the supply of electrons from water splitting by PSII (Bombelli et al., 2011; Pisciotta et al., 2011; Cereda et al., 2014), we measured the rate of oxygen evolution by wild-type or Δ*pilD*^∗^ cells. For both photoautotrophically and photoheterotrophically cultured cells, the growth rate, chlorophyll content, and oxygen evolution of the Δ*pilD*^∗^ was not significantly different to that of the wild-type organism (Table 1). This suggests that PSII activity and the photosynthetic capacity of the Δ*pilD*^∗^ strain are similar to the wild type, allowing direct electrochemical comparison of the two strains when the same number of cells is used (normalized by OD_750_).

### Electrochemical Properties of the Δ*pilD*^∗^ Strain

**FIGURE 2:**
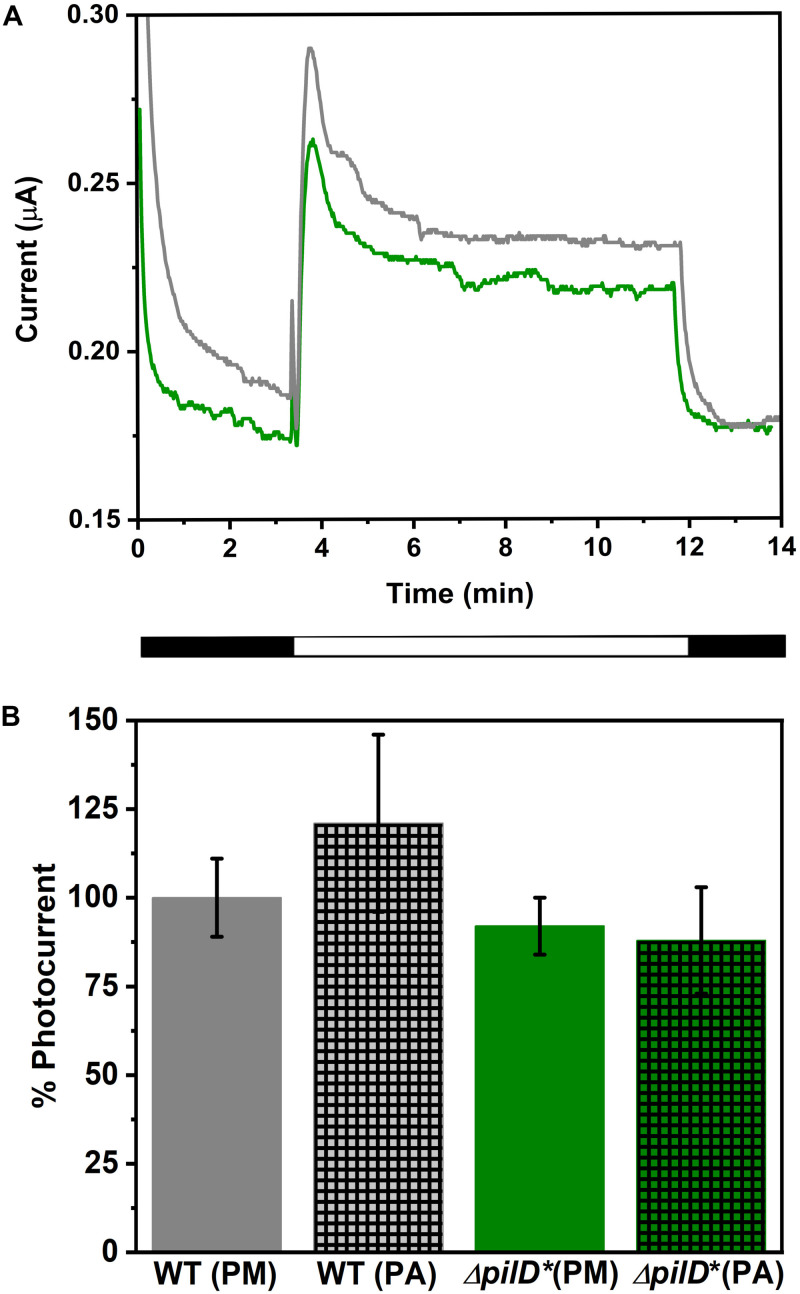
**(A)** Chronoamperograms showing photocurrent produced by wild-type (gray line) and ΔpilD* mutant (green line) *Synechocystis* immobilized on a carbon cloth electrode. Current production in the dark was allowed to stabilize prior to illumination at which point a sudden increase in current is observed. After approximately 12 min, cells are returned to the dark and a sudden decrease in current is observed. The light and dark phases are shown schematically under the *x*-axis. **(B)** Comparison of photocurrent produced by WT and ΔpilD* mutant *Synechocystis* cells. The photocurrent is normalized to the cell density of the sample applied to the working electrode, and photocurrent produced by WT grown under photomixotrophic conditions is set at 100%. Strains were grown under photomixotrophic (solid bars labeled PM) or photoautotrophic (hatched bars labeled PA) conditions (as described in “Materials and Methods”) and harvested at a similar phase of growth (determined by OD_750_). Error bars represent one standard deviation from the mean of three independent experiments.

The light-dependent, EET capacity of the wild-type and Δ*pilD*^∗^ strains of *Synechocystis* was probed by measuring the photocurrent produced when a potential of +240 mV (vs. standard hydrogen electrode) was applied. This potential was chosen because it has previously been shown to be sufficiently oxidizing for the cells to transfer electrons to an external substrate (Cereda et al., 2014). As shown in Figure 2A, when Δ*pilD*^∗^ cells are applied to the working electrode of a photo-bioelectrochemical cell followed by incubation for a few minutes at the desired electrochemical potential, photocurrent can be observed [red light with peak λ = 660 nm, maximum intensity 20 W m^–2^ (110 μmol photons m^–2^ s^–1^)]. The photocurrent produced by Δ*pilD*^∗^ is similar to the photocurrent produced by wild type whether the cells were grown photoautotrophically or photomixotrophically (Figure 2B). For the Δ*pilD*^∗^ strain, photocurrent increases linearly (*R*^2^ = 0.99) with cell density to a magnitude (88 ± 15%) comparable to that produced by the wild type (100 ± 12%) (Supplementary Figure S1). This shows that the electrical output of both strains is directly related to the concentration of *Synechocystis* cells present in the electrochemical cell. In short, photocurrent production by the two strains is not significantly different, suggesting that it is independent of Tfp.

### Atomic Force Microscopy (AFM) Imaging of Wild-Type and Δ*pilD*^∗^ Cells

**FIGURE 3:**
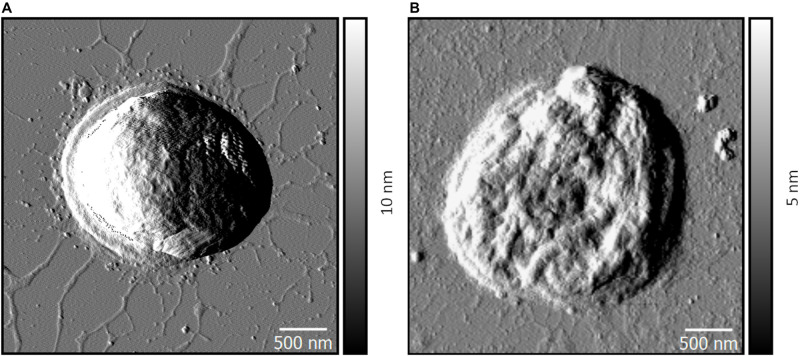
Representative AFM amplitude images of wild-type **(A)** and ΔpilD* **(B)** *Synechocystis* cells.

Planktonic growth under rapidly mixed conditions has previously been reported to negatively impact pili stability via shearing action (Yoshihara et al., 2001; Lamb et al., 2014). To provide evidence that wild-type *Synechocystis* has Tfp under the growth conditions employed in this study, we visualized the cells by AFM. To ensure that the imaged cells are as morphologically like those used in the electrochemical measurements, samples were washed in deionized water prior to AFM visualization to remove contaminants, simulating the pretreatment conditions used for the electrochemical experiments. Figure 3 shows representative images. Wild-type cells grown planktonically have hair-like pilus structures protruding from the cell surfaces (Figure 3A). Conversely, corresponding images of Δ*pilD*^∗^ cells grown and treated in the same way reveal an almost complete lack of cell surface protrusions (Figure 3B).

### Scanning Electron Microscopy (SEM) Imaging of *Synechocystis* Cells

**FIGURE 4:**
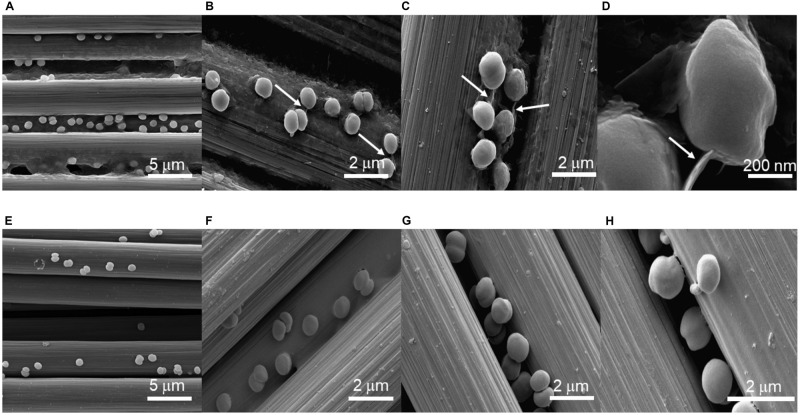
Scanning electron micrographs of wild-type **(A–D)** and **Δ**pilD* **(E–H)** *Synechocystis* cells immobilized on a carbon cloth electrode. Arrows in panels **(B–D)** point to structures consistent with pili.

Scanning electron microscopy was used to visualize the physical interaction between *Synechocystis* cells and the carbon electrode. SEM micrographs of both wild-type and Δ*pilD*^∗^ cells confirm uniform adhesion of cells to the carbon cloth electrode surface. We note that sample preparation for SEM imaging can affect the total number of cells attached to the electrode and can underestimate the actual coverage. Nonetheless, in all images, cells appear to be in direct contact with the carbon cloth electrode. High-resolution images from wild-type cells clearly show structures consistent with being pili present between the cells and the carbon substrate (Figures 4A–D). Conversely, high-resolution images from the Δ*pilD*^∗^ strain show a complete absence of any type of pilus-like structures (Figures 4E–H), suggesting some other mechanism for the physical interaction with the electrode surface.

### Conductivity Measurements of Pili Using AFM

**FIGURE 5:**
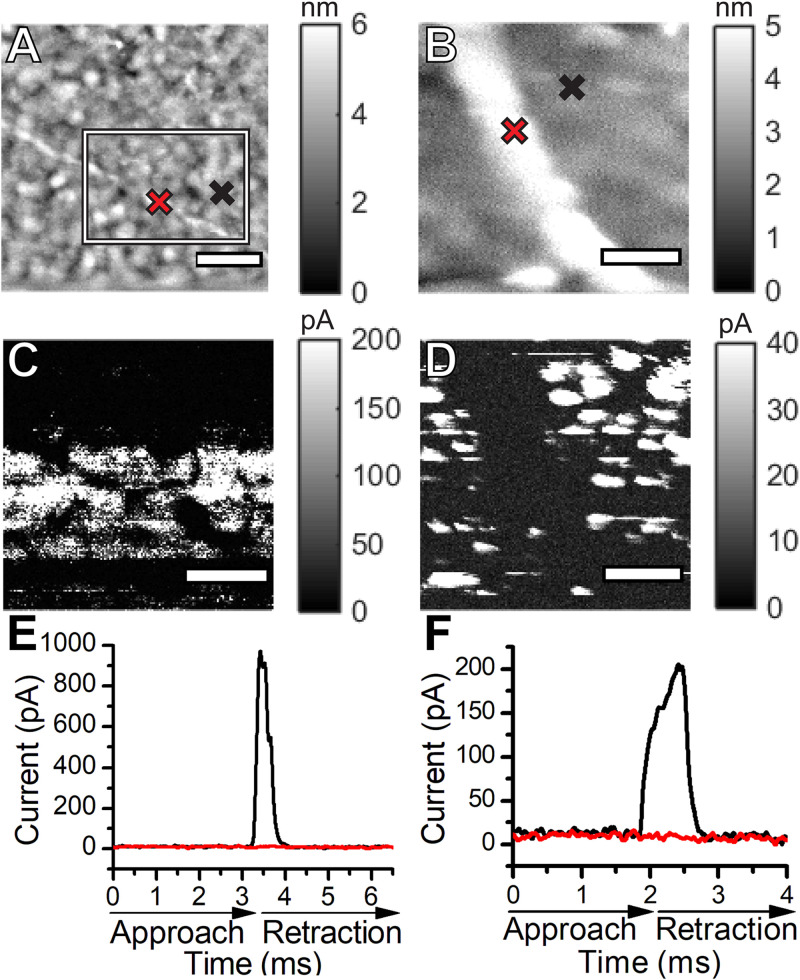
Topographical atomic force microscopy images of a thin **(A)** and a thick **(B)** *Synechocystis* pilus. Current maps of the thin **(C)** and the thick **(D)** *Synechocystis* pili shown in **(A,B)**. The current map in **(C)** shows a zoomed in region of the thin pilus, approximated by the box in **(A)**. Representative current versus time curves during probe approach and retraction over the pili (red) and over the Au substrate (black) for the thin **(E)** and thick **(F)** pili. Cross marks in the topographical images indicate the locations where the curves were measured over the pili (red) and over the Au substrates (black). The scale bars in **(A,B,D)** indicate 200 nm. The scale bar in **(C)** indicates 100 nm.

The Fast Current Mapping (FCM) mode of AFM was used to simultaneously generate topographical and current map images of *Synechocystis* pili overtop Au-coated glass coverslips. FCM was chosen for the conductivity measurements to minimize lateral tip-sample forces, which we observed to be damaging and disruptive to the filaments in contact mode conductive AFM. During FCM, current and force curves are generated at each pixel, while the AFM probe vertically approaches and retracts from the sample. Thick and thin pili are clearly visible in the topographical images (Figures 5A,B). The diameters of the thin (Figure 5A) and thick (Figure 5B) pili were obtained from AFM height measurements as 3 and 6 nm, respectively. Note that the heights, rather than the apparent widths, were used to estimate the diameters, since AFM lateral measurements are subject to tip convolution artifacts resulting in a significant broadening of structures. There are no current readings along the lengths of pili in the current map images (Figures 5C,D). Representative point measurements of current during probe approach and retraction (Figures 5E,F) show pili current readings comparable to background values when the probe contacts the pili with the same force used to observe current readings from the Au substrate. Our results indicate that, within the sensitivity of our instrumentation, *Synechocystis* pili are not conductive. We note that AFM measurements were made with dried cells and conductivity may differ under other conditions.

## Discussion

Conductive pili are hypothesized to be important for long-range electron transport by various microorganisms including dissimilatory metal-reducing bacteria such as *G. sulfurreducens*. Gorby et al. (2006) reported scanning tunneling microscopy images suggesting that, under CO_2_ limitation, *Synechocystis* also produces such conductive filaments. However, controversy exists as to whether the structures they observed are true Tfp assemblies. Lovley (2012) has suggested the diameter of the filaments is too large for Tfp. Furthermore, it is hypothesized that similar structures observed in *S. oneidensis* by Gorby et al. (2006) in the same study are filamentous extracellular polysaccharides that arise as an artifact of dehydration during sample preparation or imaging (Dohnalkova et al., 2011). Finally, although appendages produced by *S. oneidensis* have been shown to be conductive under dry conditions (Gorby et al., 2006; El-Naggar et al., 2010), additional work has shown that nanowires of *S. oneidensis* MR-1 are not pili but rather outer membrane extensions containing the multiheme cytochrome conduits of EET (Pirbadian et al., 2014). Consistent with these findings, experiments with mutant strains of *S. oneidensis* have shown that pili are not required for EET (Bouhenni et al., 2010). Thus, the potential role of pili in EET in cyanobacteria such as *Synechocystis* was ambiguous and warranted investigation.

The results herein show that our Δ*pilD*^∗^ strain, which lacks the *pilD* gene and is unable to synthesize mature pili, produces a similar amount of light-dependent current as wild-type *Synechocystis* in a mediatorless biophotovoltaic device. Given that the rate of photo-electron production by PSII was shown to be similar in the mutant and wild-type using oxygen evolution measurements, we conclude that, at least under the conditions used in this study, pili are not required for photocurrent production. In support of this conclusion, our AFM-based electrical measurements suggest that neither thick nor thin pili of *Synechocystis* are conductive. Microbial cell-to-electrode electron transfer by *Synechocystis* must therefore be facilitated by an alternative, i.e., non-pili-mediated, mechanism, either by direct transfer from some other cell surface electron transport proteins or by mediated-transfer via unknown redox-shuttles excreted into the extracellular environment/electrolyte (Saper et al., 2018; Wenzel et al., 2018). Secreted flavins have been detected in cultures of *Shewanella* and other bacteria and are believed to play a role in EET by serving as soluble redox mediators (Okamoto et al., 2013; Tian et al., 2019).

We confirmed direct contact between *Synechocystis* cells and the carbon cloth electrode with high-resolution SEM images. This demonstrates that the absence of pili in the Δ*pilD*^∗^ mutant cells does not appear to affect the adhesion of the mutant cells to the electrode surface, and mediated electron transfer may be more important in cyanobacteria than electron transfer via direct contact between cells and the electrode. Wenzel et al. (2018) elegantly demonstrated that bio-anodes with mesopores large enough to accommodate cells, thereby providing an increase in the direct contact area between the bacteria and the electrode surface, showed only a small increase in current generation compared to nanoporous electrodes, which are not directly accessible to the relatively large cells but provide an increased surface area for interactions with soluble redox-carriers. Coupled with our demonstration that pili do not appear to be necessary for EET, it appears most likely that cyanobacteria use a redox shuttle-mediated mechanism for electron transfer from the bacteria to the electrode rather than a direct electron transfer, or both mechanisms may be important under different growth conditions or environmental stresses. Identifying the components responsible for the reduction of the extracellular environment by cyanobacteria is a crucial next step, both for exploiting cyanobacterial EET and determining the role of this phenomenon in natural systems.

## Data Availability Statement

The raw data supporting the conclusions of this article will be made available by the authors, without undue reservation.

## Author Contributions

JH, TB, and AJ conceived the study and designed the research. AH and JH generated and characterized the Δ*pilD* mutant. MT, JL, BD, and RR performed or analyzed the atomic force microscopy. MT and AJ performed or analyzed the scanning electron microscopy. MC and ME-N performed conductive AFM. MT, AC, and AJ performed or analyzed the electrochemical experiments. MT, AH, JH, TB, and AJ wrote the manuscript, which was edited and approved for submission by all the other authors.

## Conflict of Interest

The authors declare that the research was conducted in the absence of any commercial or financial relationships that could be construed as a potential conflict of interest.
