# Supplementary material for: Type IV Pili-Independent Photocurrent Production by the Cyanobacterium Synechocystis sp. PCC 6803
Source: Front Microbiol. 2020 Jun 25;11:1344. doi: 10.3389/fmicb.2020.01344 (PMC7344198; doi:10.3389/fmicb.2020.01344)
Supplement: Supplementary file 1 [file Data_Sheet_1.PDF]

Supplementary Material

Supplementary Table 1. Strains of *Synechocystis* sp. PCC 6803 used in this study

Strain	Description	Source/Reference
Wild-type (WT)	Glucose tolerant strain of *Synechocystis* sp. PCC6803 (Williams, 1988)[a]	Nixon Lab, Imperial College, London, UK
Δ*psbB*	*psbB* (slr0906) deletion mutant; zeocin resistant	Cereda *et al*. 2014[b]
Δ*pilD*	*pilD* (slr1120) deletion mutant; chloramphenicol resistant (CmR)	This study
Δ*pilD**	Unknown suppressor mutation of Δ*pilD* capable of photoautotrophic growth; CmR	This study

[a] Williams J.G.K. (1988) Construction of Specific Mutations in Photosystem II Photosynthetic Reaction Center by Genetic-Engineering Methods in *Synechocystis* 6803. *Method. Enzymol*. **167**, 766-78.

[b] Cereda, A., Hitchcock, A., Symes, M.D., Cronin, L., Bibby, T.S., and Jones, A.K. (2014) A bioelectrochemical approach to characterize extracellular electron transfer by *Synechocystis* sp. PCC6803. *PloS One*. **9**, e91484.

Supplementary Table 2. Oligonucleotides used in this study

Primer	Sequence (5'-3')
pilD-us-F	CGACGTTGTAAAACGACGGCCAGTGAATTCGCG ATCGCCAAGCTGTTA
pilD-us-R	CGCCAAAAGTTGGCCCAGGGCTTCCCGGTATTA TTAGGCCACCACGTTGAGAAAACT
cat-F	GCCGTGGGTAGTTTTCTCAACGTGGTGGCCTAA TAATACCGGGAAGCCCTGGGCCAACTTTTGGCG
cat-R	TTGTGGGGTCACAAAATTTAAGTAACTAGTTTA CGCCCCGCCCTGCCACTCATCGCAGTA
pilD-ds-F	TACTGCGATGAGTGGCAGGGCGGGGCGTAAAC TAGTTACTTAAATTTTGTGACCCCACAA
pilD-ds-R	AACAGCTATGACCATGATTACGCCAAGCTTGTC AGGGTTGGATTTTGGAAGGTTATCCTC
pilD-screen-F	TCGACAAAAGACGGTTTGAA
pilD-screen-R	GCTCTAACAATACCGGGGATA
pilD-RT-F	GACCCTGCCCAATAGTTTGA
pilD-RT-R	TCCCTACCGAGCAACAGACT
pilA1-RT-F	AAAACGGGCAGAAGGTGGTT
pilA1-RT-R	TTCGGACTCCCGTGCTTTAC
rnpB-RT-F	CAAACTTGCTGGGTAACGCC
rnpB-RT-R	TACTGCTGGTGCGCTCTTAC

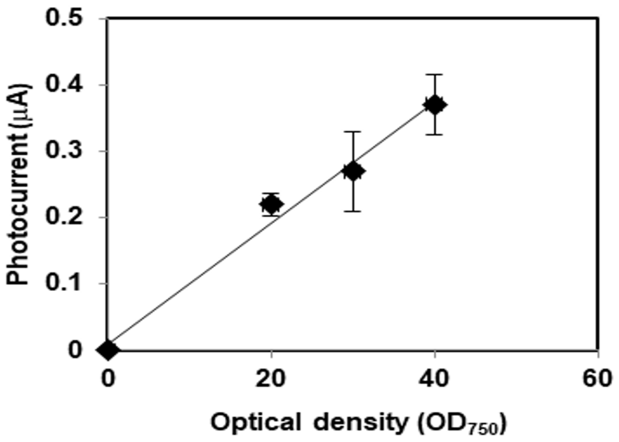

Supplementary Figure 1. Photocurrent in a mediatorless biophotovoltaic device depends linearly on concentration of Δ*pilD* Synechocystis* cells present in the electrochemical experiments. The solid line is the line of best fit through the data with a correlation coefficient of $R^2 = 0.9854$. The error bars correspond to the uncertainty in both current and cell densities.
